# Biocomputing and Synthetic Biology in Cells: Cells Special Issue

**DOI:** 10.3390/cells9112459

**Published:** 2020-11-11

**Authors:** Feifei Cui, Quan Zou

**Affiliations:** 1 Institute of Fundamental and Frontier Sciences, University of Electronic Science and Technology of China, Chengdu 610054, China; feifeicui1987@126.com; 2 Yangtze Delta Region Institute (Quzhou), University of Electronic Science and Technology of China, Quzhou 324000, China; 3 Hainan Key Laboratory for Computational Science and Application, Hainan Normal University, Haikou 570206, China

Biocomputing and synthetic biology have been two of the most exciting emerging fields in recent years. Biocomputing focuses on developing novel computational models beyond the Turing machine, such as DNA computing and membrane computing. It aims at creating a super machine in cells without any silicon. Synthetic biology, a more detailed extension of biocomputing, involves the design of circuits, simulations and cell analysis. It is interdisciplinary, involving the chemical industry, biotechnology, computer science and mathematics.

This Special Issue aims at bringing together researchers to disseminate their novel computational techniques in biocomputing and synthetic biology, particularly those involving interdisciplinary research. This Special Issue contains 13 research papers addressing the computational techniques for membrane protein prediction, DNA related site identification, association identification between microRNA and diseases, and gene interaction detection. A brief description of each of accepted articles is as follows. 

The article by Ye et al. [1] presents a framework for the detection of the interactive gene groups for scRNA-seq data based on co-expression network analysis and subgraph learning. A gene co-expression network is constructed by using the differentially expressed genes of each cell subpopulation, identified via the use of spectral clustering. Then, in the constructed gene co-expression network, the dense subgraphs are learnt to detect the interactive gene groups. A real cancer scRNA-seq dataset is used for testing the proposed framework to detect interactive gene groups of different cancer subtypes, and results show that different gene co-expression networks and interactive gene groups with different functional enrichment are presented in different subtypes.

The article by Tao et al. [2] implements the systematic identification of housekeeping genes (HKG) used as references in *Caenorhabditis elegans* from the large-scale datasets by an unbiased genome-wide search, in order to study the problems of accurate gene expression quantification. A total of 145 microarray datasets are used for perforing the unbiased identification of reference genes in *Caenorhabditis elegans*. Six algorithms are utilized for normalizing raw data to obtain a gene expression matrix (GEM) for each microarray dataset, and first-round ranked gene lists (GL) that contain multiple gene lists for each algorithm are achieved based on gene expression stability. Then, the second-round ranked gene lists (SGL), that contain one gene list for each of the six algorithms are produced using the *RankAggreg* algorithm. Finally, the final reliable HKG candidates are identified by intersecting the produced six SGLs.

Two articles focus on the identification of DNA4-methylcytosine sites based on computational approaches. Work by Wahab et al. [3] proposes a deep learning based computational model, called DNC4mC-Deep, for identifying the DNA4-methylcytosine sites in the genomes of *F. vesca*, *R. chinensis*, and a cross-species dataset. Several different feature encoding schemes are used for representing DNA sequences, including: binary encoding (BE), Kmer-based encoding (di-nucleotide composition (DNC) and tri-nucleotide composition (TNC), nucleotide chemical properties (NCP, contain a ring structure, hydrogen bond and functional group), the nucleotide chemical properties with the frequencies of each nucleotide position (NCPNF) and multivariate mutual information (MMI). The encoded DNA sequence that is represented as a fixed length of feature vector is input into the identification model based on a convolutional neural network (CNN). While the article by Manavalan et al. [4] presents an ensemble learning framework for identification of DNA4-methylcytosine sites in the mouse genome. In this framework, four different machine learning algorithms and seven feature encoding schemes are employed to generate 28 probabilistic features. Specifically, features are extracted by seven feature encoding schemes, including: binary profile (BPF), electron–ion interaction pseudopotentials (EIIP) of trinucleotides, a combination of dinucleotide binary encoding (DPE), and local position-specific dinucleotide frequency (LPDF) represented as M6AMRFS, k-mer composition (Kmer), ring-function-hydrogen-chemical properties (RFHC), dinucleotide-(DPCP) and trinucleotide-physicochemical properties (TPCP). Then, extracted features are used for four machine learning algorithms, namely gradient boosting (GB), extremely randomized tree (ERT), support vector machine (SVM) and random forest (RF). Then, these probabilistic features are used as a feature vector and once again input into machine learning algorithms to form the ensemble classifier that can be used for identifying the DNA4-methylcytosine sites containing samples, from those not containing DNA4-methylcytosine sites. Moreover, a user-friendly web server, namely 4mCpred-EL is provided.

The article by Wang et al. [5] implements a computational technique based on DNA methylation analysis for the detection of breast cancer (BRCA) invasiveness. Specific CpG sites in samples are identified by using two differential methylation analysis methods, namely, Empirical Bayes (EB) and significance analysis of microarrays (SAM). Next, redundant feature sites are filtered by dimensionality reduction, and then, a methylation-based classifier based on the random forest algorithm is constructed to classify primary BRCA as either invasive or noninvasive. The credibility of the classifier is validated using the Cancer Genome Atlas (TCGA) database and is also compared with the accompanying clinical data. A website for the prediction of invasiveness of BRCA, namely BMMP (BRCA Methylation Metastasis Prediction) [6], is available.

The research by Tayara et al. [7] introduces a novel computational model, namely DQDNN (DNA sequence Quantifying based on Deep Neural Networks), for quantifying the function of non-coding DNA regions using deep learning. In the deep neural networks-based model, convolution layers and recurrent layers are combined for capturing regularity motifs at multiple scales and capturing long term dependencies between the captured motifs, respectively. Moreover, evolutionary information is integrated with raw genomics sequences, and is proven to be a useful method for improving the model performance.

Work by Tan et al. [8] proposes a model ensemble of classifiers for the identification of enhancers using deep learning methods. The sample data are represented by six di-nucleotide physicochemical properties, including rise, roll, shift, slide, tilt and twist, the value of each property is normalized to a range of zero to one. Each di-nucleotide is converted to a vector of size six, and then, vector represented di-nucleotides of the sample data are used as inputs to the deep neural networks-based classifier. In the classifier model, convolution and max pooling layers are used before bi-directional recurrent and fully-connected layers. The results show that the deep model has the potential for improving the performance of shallow machine learning approaches.

Three articles focus on computational approaches and analyses for the identification of related binding proteins, such as phosphorylation sites in phosphoprotein-binding domains, ubiquinone-binding proteins and phage virion proteins. The article by Guo et al. [9] develops an improved framework for identification of phosphorylation sites (p-sites) that specifically interact with phosphoprotein-binding domains (PPBDs), based on deep learning methods. A framework of seven-layer deep neural networks (DNNs) is implemented to train a general model to predict PPBD-specific binding p-sites (PBSs), containing an input layer, five fully connected layers (hidden layers) and an output layer. An online service named GPS-PBS (Group-based Prediction System for identifying PBSs) is provided. Work by Lu et al. [10] is the first to propose a ubiquinone-binding proteins (UBPs) predictor, called UBPs-Pred. The optimal features are selected from three categories of sequence-derived features, including amino acid composition (AAC), dipeptide composition (DC) and a position-specific scoring matrix (PPSM). Then, the selected features are input into the extreme gradient boosting (XGBoost) classifier. Several bioinformatics methods are used for analyzing the results of the UBPs predictor, including the statistics of the binding domain motifs and protein distribution, as well as an enrichment analysis of the gene ontology (GO) and the Kyoto Encyclopedia of Genes and Genomes (KEGG) pathway, through these analyses it is found that most UBPs are membrane proteins, several motifs in the ubiquinone-binding domains are considered statistically significant, and there are many significant pathways in the KEGG pathway of human UBPs. The article by Charoenkwan et al. [11] proposes a novel computational model, namely PVPred-SCM (Phage Virion Proteins Predictor based on Scoring Card Method), for identifying phage (or bacteriophage) virion proteins (PVPs) using a scoring card method (SCM). Sequence features of propensity scores of amino acids and dipeptides are used for representing sequence data, and are then utilized for the PVP predictor using an SCM model. A user-friendly web-server for identifying the likelihood of whether or not input sequences are PVPs is provided.

There are three articles that focus on the disease related prediction, containing the prediction of disease–microRNA associations and disease–drug associations. The article by Chen et al. [12] proposes a machine learning-based method for predicting disease–microRNA associations using network topology information. The information on similarity and topology in networks is used for constructing features, then, constructed features are used for training the predictor to identify potential associations. Specifically, a bilayer network is first constructed using different integrated data sources. Then, two kinds of feature vectors, including similarity features and network embedding features guided by the DeepWalk algorithm [13], are generated. Finally, a prediction score is calculated using two types of obtained feature vectors on the deep forest model, to predict the association between diseases and microRNA. Work by Li et al. [14] also proposes the prediction model for disease–microRNA associations, but they utilize the heterogeneous graph convolutional networks. This model, termed heterogeneous graph convolutional network for miRNA–disease associations (HGCNMDA), integrates the known human protein–protein interactions (PPI) and four biological networks, including miRNA–disease, miRNA–gene, disease–gene and PPI networks. Graphs are directly input to the end-to-end deep learning architecture model, and the multi-scale characteristics of vertexes between different integrated networks. The article by Xuan et al. [15] proposes a deep learning-based method for predicting drug–disease associations. The prediction model, namely CBPred (CNN and BiLSTM prediction model), is based on a convolutional neural network (CNN) and bidirectional long short-term memory (BiLSTM), used to deeply integrate similarities and associations between drugs and diseases, and paths among drug–disease pairs. Specifically, a drug–disease heterogeneous network is constructed based on the similarities and known associations between nodes at first. The original information and topological information among nodes are then integrated using CNN and BiLSTM and are then used for obtaining deep representations and providing candidate diseases. In addition, an attention mechanism at the path level is constructed because of different paths having discriminate contributions to the association prediction.

The 13 publications in this Special Issue summarize the novel computational techniques emerging in biocomputing and synthetic biology, containing DNA computing, related protein identifying, neural computing, and gene expression detecting and quantifying. Importantly, these publications provide future research directions or potential interdisciplinary research topics within the topics of biocomputing and synthetic biology. I wish to thank all of the authors for their contributions, the scientific communities for peer reviewing, and the staff at the Cells editorial office for their work on this Special Issue.
